# Effectiveness of 13-valent pneumococcal conjugate vaccine against hypoxic pneumonia and hospitalisation in Eastern Highlands Province, Papua New Guinea: An observational cohort study

**DOI:** 10.1016/j.lanwpc.2022.100432

**Published:** 2022-03-15

**Authors:** Christopher C Blyth, Kathryn J Britton, Cattram D Nguyen, Joycelyn Sapura, John Kave, Birunu Nivio, Jocelyn Chan, Catherine Satzke, Rebecca Ford, Wendy Kirarock, Deborah Lehmann, William Pomat, Fiona M Russell

**Affiliations:** a Wesfarmers Centre for Vaccines and Infectious Diseases, Telethon Kids Institute and School of Medicine, The University of Western Australia, Nedlands, WA, Australia; b Department of Infectious Diseases, Perth Children's Hospital, Nedlands, WA, Australia; c Department of Microbiology, PathWest Laboratory Medicine, QEII Medical Centre, Nedlands WA, Australia; d Infection and Immunity, Murdoch Children's Research Institute, Melbourne, Victoria, Australia; e Department of Paediatrics, The University of Melbourne, Melbourne, Victoria, Australia; f Infection and Immunity Unit, Papua New Guinea Institute of Medical Research, Goroka, Eastern Highlands, Papua New Guinea; g Department of Microbiology and Immunology, The University of Melbourne at the Peter Doherty Institute for Infection and Immunity, Melbourne, Victoria, Australia; h Centre for International Child Health, Department of Paediatrics, The University of Melbourne, Melbourne, Victoria, Australia

**Keywords:** Pneumonia, Vaccine, *Streptococcus pneumoniae*, Infant, Child

## Abstract

**Background:**

Pneumonia is a leading cause of childhood mortality with *Streptococcus pneumoniae* a major contributor. Pneumococcal conjugate vaccines (PCVs) have been introduced into immunisation programs in many low- to middle-income countries (LMICs) yet there is a paucity of data evaluating the effectiveness in these settings. We assess the effectiveness of 13-valent PCV (13vPCV) against hypoxic pneumonia, hospitalisation and other clinical endpoints in children <5 years living in Eastern Highlands Province, Papua New Guinea (PNG).

**Methods:**

Data from two consecutive prospective observational studies (2013–2019) enrolling children <60 months presenting with pneumonia were included. Hypoxic pneumonia was defined as oxygen saturations <90%. Outcomes included hospitalisation, severe clinical pneumonia and death. 13vPCV status was determined using written records. Logistic regression models were used to estimate the odds ratios of key outcomes by 13vPCV vaccination status adjusted for confounders using inverse probability of treatment weighting.

**Findings:**

Data from 2067 children (median age; 9 months [IQR: 5–11]) were included. 739 children (36.1%) were hypoxic and 623 (30.4%) hospitalised. Twelve children (0.6% of total cohort) died in hospital. 670 children (32.7%) were fully 13vPCV-vaccinated. 13vPCV vaccination was associated with a 28.7% reduction (95% confidence interval [CI]: 9.9; 43.6%) in hypoxic pneumonia and a 57.4% reduction (38.0; 70.7%) in pneumonia hospitalisation.

**Interpretation:**

13vPCV vaccination is effective against hypoxic pneumonia and pneumonia hospitalisation in PNG children. Strategies to improve access to and coverage of 13vPCV in PNG and other similar LMICs are urgently required.

**Funding:**

Funded by Pfizer Global and the Bill & Melinda Gates Foundation.

**Research in context**

**Evidence before this study**

We searched Medline (Ovid), Embase (Ovid) and Pubmed for reports published before November 2021 for terms: pneumococcal conjugate vaccine, pneumonia, pneumo*, Streptococcus pneumoniae. We searched for studies evaluating vaccine efficacy and vaccine effectiveness (VE) of the 13-valent pneumococcal conjugate vaccine (13vPCV) against outcomes of hypoxic pneumonia and mortality attributable to pneumonia. The population of interest was children under five years of age, using either randomised control trials (RCTs), observational population-based or case-control studies. We identified three studied that identified a 23-72% reduction in hypoxic pneumonia post 13vPCV introduction. Four observational studies reported VE of PCV between 8-71% against pneumonia-related mortality. Only one study was conducted in the Asia-Pacific region.

**Added value of this study**

Between January 2013 and December 2019, we conducted a prospective observational study in the highlands of Papua New Guinea (PNG) to determine the 13vPCV VE against hypoxic pneumonia, hospitalisation and other clinical endpoints in children <5 years. Enrolling 2067 children, we found that 13vPCV was associated with a 28.7% reduction (95% confidence interval [CI]: 9.9; 43.6%) in hypoxic pneumonia and a 57.4% reduction (38.0; 70.7%) in pneumonia hospitalisation. This study demonstrates that 13vPCV vaccination is effective against hypoxic pneumonia and pneumonia hospitalisation in PNG children. Effectiveness against in-hospital mortality could not be demonstrated.

**Implications of all the available evidence**

This study highlights the tremendous benefits of PCVs in childhood immunisation programs. Given the ongoing high burden of pneumonia and challenges in seeking health care in PNG, routine use of 13vPCV is expected to substantially reduce the burden of pneumonia. These data provide the evidence required to support policy makers’ decisions with regards to inclusion in the immunisation schedule, promotion and use of 13vPCV in PNG and other low- and middle-income countries. Given inadequate vaccine coverage, ongoing investment in the national immunisation program and strengthening of health systems must be prioritised, particularly during the COVID-19 pandemic, if the expected benefits of 13vPCV and other childhood vaccines are to be realised.

Alt-text: Unlabelled box

## Introduction

Lower respiratory infection including pneumonia remains a major cause of morbidity and mortality in children.^1^ Although a broad range of viral and bacterial pathogens contribute to this burden, *Streptococcus pneumoniae* remains a major contributor to pneumonia-related mortality.^1^ A small number of randomised controlled trials of pneumococcal conjugate vaccines (PCVs) and observational studies have confirmed efficacy and effectiveness against radiologically-confirmed childhood pneumonia and pneumonia-related mortality.^2^, ^3^, ^4^, ^5^, ^6^ Evaluations of PCV in The Gambia^7^ and Malawi^8^ have demonstrated a positive impact against clinical and hypoxic pneumonia, a precursor of mortality. Most recently, a study in Lao People's Democratic Republic^9^ has demonstrated effectiveness against hypoxic pneumonia.

Given the ongoing burden of disease and demonstrated impact, PCVs have been included in routine childhood schedules in numerous countries, including most recently, a number of low- to middle-income countries (LMICs) in Asia and the Pacific. The World Health Organization (WHO) recommends that the use of PCVs is evaluated to inform vaccine policy. Despite this recommendation, there remains a paucity of published data, particularly following introduction of PCVs into Asia and the Pacific.^9^

Papua New Guinea (PNG) has been previously identified as a high priority country for the achievement of the Sustainable Development Goals because the baseline child mortality rate was among the highest in the Western Pacific Region (<5 year old mortality rate in 2015: 57 per 1000 live births^10^). Pneumonia is the most common cause for childhood death in PNG.^11^^,^^12^ Studies conducted in the highlands of PNG have repeatedly demonstrated the importance of *S. pneumoniae* in the aetiology of pneumonia.^13^, ^14^, ^15^, ^16^, ^17^ Most recently, of pneumonia and meningitis episodes with proven bacteraemia (4% of total), *S. pneumoniae* was the infecting pathogen in 53% of cases.^17^ The findings prompted the introduction of pneumococcal conjugate vaccine into the routine PNG infant vaccination schedule. Prevenar13 (Pfizer; 13vPCV) was chosen for this purpose. From 2014, PNG infants were recommended to receive 13vPCV at one, two and three months of age (as a 3+0 schedule). However, because of the broad range of circulating pneumococcal serotypes, it was not known how effective 13vPCV would be against pneumonia in this high burden setting.

Given the challenges of demonstrating PCV effectiveness in LMICs, we sought to determine the effectiveness of 13vPCV introduction into the highlands of PNG using data from two observational studies. Using methods described previously,^9^ we sought to determine vaccine effectiveness (VE) against hypoxic pneumonia, pneumonia hospitalisation, severe clinical pneumonia, and death in PNG children <5 years of age.

## Methods

The study was conducted in the Eastern Highlands Province (EHP) of PNG which has a population of 579,825 (2011 census).^18^ Goroka, the capital of EHP is located 1600 m above sea-level, is only accessible by air from the national capital, Port Moresby and by a narrow mountainous highway from the nearest sea port, Lae. A full description of the study population, health facilities and socioeconomic factors has been previously published.^19^

The national 13vPCV program commenced in 2014 with official national three dose vaccine coverage increasing from 20% in 2015 to 38% in 2019.^20^ 13vPCV was provided in addition to vaccines already on the PNG schedule: neonatal BCG and hepatitis B vaccines followed by three doses of diphtheria, tetanus, whole-cell pertussis, hepatitis B and *Haemophilus influenzae* type b vaccine (DTPw-HepB-Hib) and oral polio vaccines. Routine measles vaccines were recommended at 6 and 9 months and subsequently changed in 2015 to measles-rubella vaccines at 6, 9 and 18 months.^21^

This analysis uses data from two studies conducted at Eastern Highlands Provincial Hospital (EHPH, previously known as the Goroka General Hospital, GGH), the referral hospital servicing the whole Eastern Highlands Province and community healthcare clinics within Goroka town.

All children enrolled in the two studies and therefore included in this analysis, fulfilled the same eligibility requirements for enrolment (i.e. PNG-defined moderate or severe pneumonia). The first study commenced in January 2013 prior to the national 13vPCV introduction and recruited clinical pneumonia cases presenting to EHPH or a community healthcare clinic.^17^^,^^19^ The original aim of this study, which finished recruitment in January 2016, was to determine the aetiology of moderate and severe pneumonia^21^ (and/or meningitis). The second study, commencing in January 2016, recruited clinical pneumonia cases from EHPH and community clinics and conducted household community surveys to determine community vaccine uptake. This study was part of a multi-country evaluation of 13vPCV (Laos, Mongolia, PNG) to explore the PCV coverage required to observe indirect effects by assessing the impact of PCV on nasopharyngeal pneumococcal colonisation in vaccinated and under-vaccinated children.^22^ Children recruited up to December 2019 were included in the analysis.

### Study participants

Children <5 years of age presenting for health care (January 2013 to December 2019) with moderate or severe pneumonia, as described in the PNG clinical manual,^21^ were eligible for inclusion. As per these guidelines, PNG-defined moderate pneumonia was identified clinically by the presence of cough, tachypnoea (≥60/min if <2 months, ≥40/min if ≥2 months of age) and lower chest wall indrawing. Severe pneumonia was identified by the presence of PNG-defined moderate pneumonia with one of the following features: (i) Pulse >160/min with hepatomegaly >2 cm below costal margin; (ii) cyanosis or restlessness or (iii) inability to breastfeed/drink or vomiting.

### Study procedures

Eligible children were identified by clinically-trained research staff using screening questionnaires. Upon determining eligibility and obtaining informed consent, a brief history and examination was undertaken by research staff before laboratory specimens were obtained and treatment provided. Verbal consent and minimal data were obtained initially ensuring timely provision of appropriate medical care. Following written consent, research staff conducted a more in-depth interview documenting symptoms, potential risk factors, recent antibiotic exposure and a full clinical examination including pulse oximetry. Individual handheld health records were examined for perinatal history, co-morbidities and vaccination status. From 2016, additional data including breastfeeding, dwelling type, number of household residents and household wood and cigarette smoke exposure were sought.

Children deemed well enough to be managed as outpatients were discharged and reviewed within 48 h. Pneumonia treatment was as per the PNG clinical manual.^21^ All hospitalised cases were reassessed daily whilst inpatients by clinically-trained research staff. Following discharge, we sought to follow up all children in the community within 4 weeks of discharge. Data collection forms of all participants were reviewed and uploaded into a FileMaker Pro (Claris, CA, USA) database. Data cleaning and analysis were conducted in Stata 15.^23^

### Study measures

As there is no gold standard case definition for childhood pneumonia,^24^ we used four distinct pneumonia outcomes. The primary outcome, hypoxic pneumonia, was defined as an oxygen saturation of <90% in a child with pneumonia.^9^ Oxygen saturation was recorded using the LifeBox Pulse Oximeter, Acare Technology Co., Ltd, Taiwan, preferentially in room air. If no record of saturations was obtained prior to use of supplemental oxygen, these children were assessed using their first oxygen saturation (hypoxic [i.e. saturation of <90%] or non-hypoxic). Given the potential that these children could be misclassified as non-hypoxic, sensitivity analyses were undertaken excluding those whose first oxygen saturation measurement was taken post oxygen supplementation from analysis of the primary outcome. Secondary outcomes included PNG-defined severe pneumonia (previously defined), WHO-defined severe pneumonia and hospitalised pneumonia. WHO-defined severe pneumonia (2011 definitions) was derived post-hoc using documented respiratory rate (≥50/min if <12 months, ≥40/min if ≥12 months of age) and presence of WHO-defined danger signs including: inability to drink, persistent vomiting, lethargy or loss of consciousness, stridor or severe malnutrition.^25^ Hospitalised pneumonia was that deemed severe enough to require hospital admission. Vaccination status was determined using the PNG parent/guardian held childhood record. Children were considered 13vPCV-vaccinated if they had received two doses of 13vPCV between 0 and 11 months of age; and for those children aged 12 months and older, if they had received one or more doses of 13vPCV after their first birthday.^26^ Vaccination at any time prior to presentation was considered a valid dose. Children were considered under-vaccinated if they had received fewer doses by age group. Vaccine effectiveness (VE) was estimated by comparing 13vPCV vaccination rates in both cases and controls.^27^ Cases and controls were all children in the cohort presenting with pneumonia, whilst cases were those diagnosed with the specific outcomes of interest.

### Statistical analysis

Categorical data were summarised as an absolute number and percentage of the total cohort. Continuous data were summarised as medians and interquartile ranges. Age was reported as both a continuous and categorical variable. For each study outcome (hypoxic pneumonia, severe clinical pneumonia [PNG and WHO definitions], hospitalised pneumonia, in-hospital mortality), a separate logistic regression model was used to estimate the odds ratios (ORs) and confidence intervals [CIs] for 13vPCV vaccination status. The ORs were then converted to a measures of VE using the formula: VE = (1-OR)×100. To adjust for potential confounders, we used inverse probability of treatment weighting (IPTW), which weights individuals by the inverse of the probability of 13vPCV vaccination conditional on covariates (i.e. the propensity score (PS)).^9^ The PS was estimated using a logistic regression model with 13vPCV status as the outcome variable and the following covariates: age (as a continuous variable); sex; year of enrolment; season (wet [December to April] or dry [May to November]); distance to health care (<1 h; ≥1 h); comorbid conditions (previously diagnosed conditions identified at the time of hospital presentation; assessed by parental report and review of each child's handheld record) and malnutrition (weight-for-age-z-score <2 standard deviations below mean).  To assess balance of covariates between vaccinated and under-vaccinated groups, we calculated standardised differences for each covariate before and after weighting. Covariates were considered balanced if the weighted standardised differences were <10%. For comparison, we also used a more traditional approach, fitting a multivariable logistic regression model with adjustment for confounders. Sensitivity analyses were conducted by excluding those who were reported to be non-hypoxic (i.e. saturations 90% and above) yet received supplementary oxygen and excluding years of low vaccine uptake (defined as <5% coverage).

### Sample size calculation

We estimated that the 13vPCV VE against hypoxic pneumonia would be 40% based upon a published study.^7^ Assuming 30% of under-vaccinated cases had hypoxic pneumonia, a sample size of 270 vaccinated and 270 under-vaccinated cases would estimate a VE of 40% with 95% confidence intervals from 11 to 60%.

### Ethics approval

The study was conducted in accordance with the STROBE statement (Supplemental table 1). This studies was conducted according to the study protocol approved by the Papua New Guinea Institute of Medical Research Institutional Review Board (IRB#1510), PNG Medical Research Advisory Committee (MRAC 15.19/16.09), The Royal Children's Hospital Human Resources Ethics Committee (HREC reference number:35249) and University of Western Australia Human Research Ethics Committee (RA/4/1/7960).

### Role of the funding source

Study 1 were funded by Pfizer Global and study 2 by the Bill & Melinda Gates Foundation (OPP1115490). The funder had no role in study design, data collection, data analysis, data interpretation, or writing of the report. The corresponding author had full access to all data in the study and had final responsibility for the decision to submit for publication.

## Results

**Figure 1:**
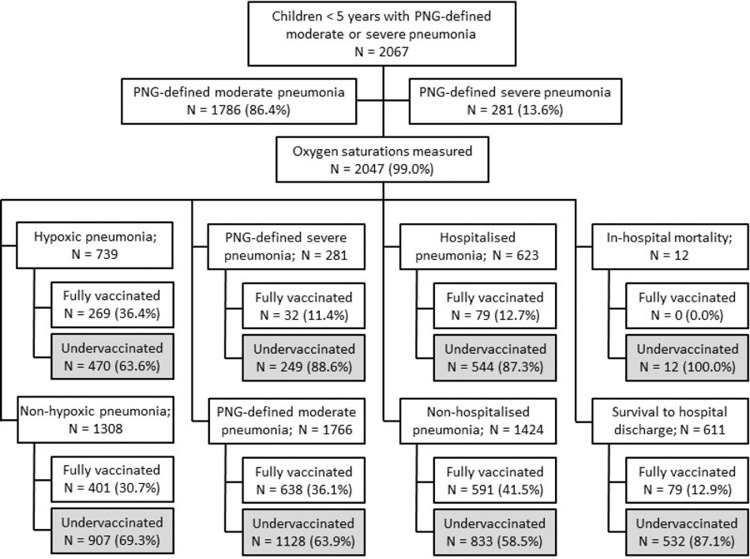
Study profile: Pneumonia outcomes by vaccination status. Clinical outcomes (hypoxic pneumonia; PNG-defined severe pneumonia; Hospitalised pneumonia and in-hospital mortality) are not mutually exclusive.

**Figure 2:**
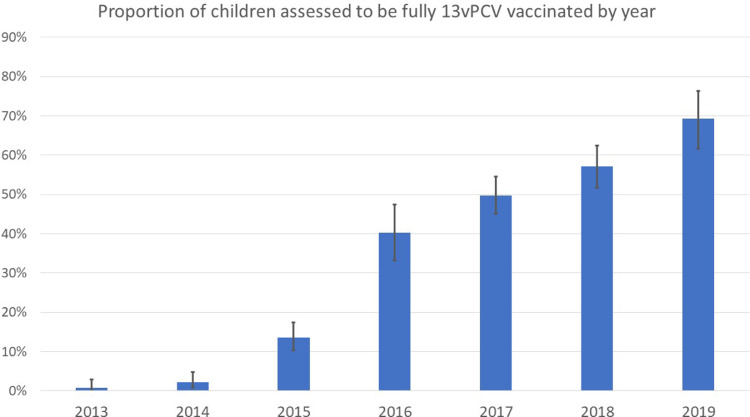
Percentage of children presenting with PNG-defined moderate and/or severe pneumonia who were considered fully 13vPCV-vaccinated.^1^

Between January 2013 and December 2019, a total of 2067 children were enrolled with 281 (13.6%) diagnosed with PNG-defined severe pneumonia. Of the total cohort, 2047 of 2067 had oxygen saturations recorded (99.0%) and were included in the final analysis (Figure 1; Table 1). Of these, 739 were hypoxic on presentation (36.1%). In addition to hypoxic pneumonia and PNG-defined severe pneumonia, other key outcomes assessed included WHO-defined severe pneumonia (*n*=724; 35.4%), pneumonia hospitalisation (*n*=623; 30.4%) and in-hospital mortality (*n*=12; 0.6% of the total cohort). Immunisation histories were available on all enrolled children. Of 2047 children included in the analysis, 670 (32.7%) were considered fully 13vPCV-vaccinated. The percentage of vaccinated children increased throughout the study period (Figure 2). The median age was 9 months (interquartile range; 5, 17 months), 1162 (56.8%) were male and 428 (20.9%) were considered to be malnourished. Only 17 (0.8%) had diagnosed comorbidities on presentation.

**Table 1:** Characteristics of children aged 0–59 months presenting with PNG-defined moderate or severe pneumonia^21^ to the Eastern Highlands Provincial Hospital and community health-care clinics, Goroka, Papua New Guinea, January 2013 to December 2019.

Characteristics	Hypoxic pneumonia (1⁰ outcome) (*n* = 739)	PNG-defined severe pneumonia (*n* = 281)	WHO-defined severe pneumonia^1^ (*n* = 724)	Hospitalised pneumonia (*n* = 623)	In-hospital death (12)	Overall (*n* = 2047)
**Demographics**						
Median age in months, (IQR)	8 (4–13)	5 (3–9)	9 (4–17)	6 (3–11)	9 (3–11)	9 (5–17)
Age, n (%)						
<12 months	492 (66.6)	237 (84.3)	433 (59.8)	479 (76.9)	10 (83.3)	1241 (60.6)
≥12 months	247 (33.4)	44 (15.7)	291 (40.2)	144 (23.1)	2 (16.7)	806 (39.4)
Sex, n (%)						
Male	408 (55.2)	157 (55.9)	411 (56.8)	351 (56.3)	6 (50.0)	1162 (56.8)
Female	331 (44.8)	124 (44.1)	313 (43.2)	272 (43.7)	6 (50.0)	885 (43.23)
Year of enrolment, n (%)						
2013	79 (10.7)	72 (25.6)	141 (19.5)	187 (30.0)	2 (16.7)	253 (12.4)
2014	77 (10.4)	64 (22.8)	109 (15.1)	124 (19.9)	6 (50.0)	268 (13.1)
2015	107 (14.5)	65 (23.1)	137 (18.9)	129 (20.7)	2 (16.7)	375 (18.3)
2016	72 (9.7)	32 (11.4)	73 (10.1)	78 (12.5)	1 (8.3)	194 (9.5)
2017	152 (20.6)	22 (7.8)	100 (13.8)	45 (7.2)	-	456 (22.3)
2018	128 (17.3)	21 (7.5)	85 (11.7)	46 (7.4)	1 (8.3)	341 (16.7)
2019	124 (16.8)	5 (1.8)	79 (10.9)	14 (2.2)	-	160 (7.8)
Season^2^, n (%)						
Dry	472 (63.9)	173 (61.6)	441 (60.9)	399 (64.0)	8 (66.7)	1236 (60.4)
Wet	267 (36.1)	108 (38.4)	283 (39.1)	224 (36.0)	4 (33.3)	811 (39.6)
**Risk factors**						
Comorbidity, n (%)^3^						
Yes	6 (0.8)	4 (1.4)	8 (1.1)	10 (1.6)	1 (8.3)	17 (0.8)
No	733 (99.2)	277 (98.6)	716 (98.9)	613 (98.4)	11 (91.7)	2030 (99.2)
Median weight-for-age z-score, (IQR)	-0.87 (-1.76 to 0.08)	-1.1 (-2.18 to -0.11)	-1.82 (-2.78 to -0.46)	-1.0 (-2.21 to 0.03)	-1.00 (-3.55 to -0.28)	-0.87 (-1.80 to 0.08)
Malnutrition, n (%)^4^						
Yes	165 (22.3)	79 (28.1)	340 (47.0)	181 (29.0)	5 (41.7)	428 (20.9)
No	574 (77.7)	202 (71.9)	384 (53.0)	442 (71.0)	7 (58.3)	1619 (79.1)
Distance to the hospital, n (%)						
< 1 h	541 (73.2)	144 (51.3)	467 (64.5)	332 (53.3)	4 (33.3)	1430 (69.9)
≥ 1 h	198 (26.8)	137 (48.7)	257 (35.5)	291 (46.7)	8 (66.7)	617 (30.1)
Breastfeeding, n (%)^5^						
Yes	381 (80.0)	73 (91.2)	244 (72.8)	161 (88.5)	1 (50.0)	891 (77.5)
No	95 (20.0)	7 (8.8)	91 (27.2)	21 (11.5)	1 (50.0)	258 (22.5)
Median number of other people in the household^5^, (IQR)	6 (4–8)	6 (4–8)	6 (4–8)	5 (4–7)	4.5 (4–5)	6 (4–8)
Housing, n (%)^5^						
Permanent/semi-permanent	287 (60.5)	38 (47.5)	184 (55.4)	87 (48.1)	1 (50.0)	750 (65.6)
Traditional (thatched)	187 (39.5)	42 (52.5)	148 (44.6)	94 (51.9)	1 (50.0)	393 (34.4)
Anybody smokes cigarettes in the house, n (%)^5^						
Yes	319 (67.4)	58 (72.5)	211 (63.3)	119 (65.7)	2 (100.0)	725 (63.3)
No	154 (32.6)	22 (27.5)	122 (36.7)	62 (34.3)	-	420 (36.7)
Cooking within house, n (%)^5^						
Yes	176 (37.0)	37 (46.2)	142 (42.4)	86 (47.2)	1 (50.0)	376 (32.8)
No	299 (63.0)	43 (53.8)	193 (57.6)	96 (52.7)	1 (50.0)	771 (67.2)
**Vaccination status**^6^						
13vPCV under-vaccinated, n (%)	470 (63.6)	249 (88.6)	538 (74.31)	544 (87.3)	12 (100.0)	1377 (67.3)
13vPCV fully vaccinated, n (%)	269 (36.4)	32 (11.4)	186 (25.7%)	79 (12.7)	-	670 (32.7)

IQR, interquartile range.

1 Estimated based on respiratory rate and reported presence of WHO defined danger signs.

2 Wet season refers to period from December to April inclusive, dry season refers to period from May to November inclusive.

3 Comorbidities were assessed at the time of pneumonia presentation by parental report and review of the handheld record

4 Weight-for-age z-score <2SD.

5 Only available for the years 2016–2019.

6 Fully vaccinated: 0-11 months ≥2 doses of 13vPCV; ≥12 months ≥1 dose of 13vPCV. Under-vaccinated: 0–11 months ≤1 dose of 13vPCV; ≥12 months 0 doses of 13vPCV.

A number of differences between 13vPCV-vaccinated and under-vaccinated children were noted (Table 2). Vaccinated children were older (odds of vaccination if aged >12 months: 1.26 [1.04, 1.52]), more often currently receiving breastmilk (OR: 1.80 [1.36, 2.38]), living in permanent/semi-permanent housing (OR: 1.40 [1.10, 1.78]), and located within 1 h of the hospital (OR: 2.05 [1.66, 2.56]). Those vaccinated were less frequently malnourished (OR: 0.65 [0.51, 0.83]) and less frequently lived in family dwelling where cooking occurred inside (OR: 0.60 [0.47, 0.77]).

**Table 2:** Characteristics of children aged 0-59 months presenting with PNG-defined moderate or severe pneumonia^21^ to the Eastern Highlands Provincial Hospital and community health-care clinics, Goroka, Papua New Guinea, January 2013 to December 2019.

Characteristics	Total (*n*=2047)	13vPCV Under-vaccinated^1^ (*n*=1377)	13vPCV Fully vaccinated^1^ (*n*=670)	*p*-value^6^
**Demographics**				
Median age in months, (IQR)	9 (5–17)	8 (4–18)	10 (6–15)	<0.001
Age, n (%):				<0·02
<12 months	1241	860 (62.5)	381 (56.9)	
≥12 months	806	517 (37.5)	289 (43.1)	
Gender, n (%)				0.75
Male	1162	785 (57.0)	377 (56.3)	
Female	885	592 (43.0)	293 (43.7)	
Year of enrolment, n (%)				<0·001
2013	253	251 (18.2)	2 (0.3)^2^	
2014	268	262 (19.0)	6 (0.9)	
2015	375	324 (23.5)	51 (7.6)	
2016	194	116 (8.4)	78 (11.6)	
2017	456	229 (16.6)	227 (33.9)	
2018	341	146 (10.6)	195 (29.1)	
2019	160	49 (3.6)	111 (16.6)	
Season^3^, n (%)				0.88
Dry	1236	833 (60.5)	403 (60.1)	
Wet	811	544 (39.5)	267 (39.8)	
**Risk factors**				
Comorbidity				0.06
Yes	17	15 (1.1)	2 (0.3)	
No	2030	1362 (98.9)	668 (99.7)	
Weight-for-age z-score, (IQR)	-0.87 (-1.80 to 0.08)	-0.91 (-1.93 to 0.04)	-0.75 (-1.58 to 0.17)	<0.001
Malnutrition				<0.001
Yes	428	318 (23.1)	110 (16.4)	
No	1619	1059 (76.9)	560 (83.6)	
Distance to the hospital				<0.001
< 1 h	1430	898 (65.2)	532 (79.4)	
≥ 1 h	617	479 (34.8)	138 (20.6)	
Breastfeeding^4^				<0.001
Yes	891	388 (72.1)	503 (82.3)	
No	258	150 (27.9)	108 (17.7)	
Median number of other people in the household, (IQR)^3^	6 (4–8)	6 (4–8)	6 (4–7)	0.34
Housing^4^				0.007
Permanent/Semi permanent	750	328 (43.7%)	422 (56.3%)	
Traditional (thatched)	393	205 (52.2%)	188 (47.8%)	
Anybody smokes in house^4^				0.49
Yes	725	345 (64.4)	380 (62.4)	
No	420	191 (35.6)	229 (37.6)	
Cooking within house^4^				<0.001
Yes	376	208 (38.8)	168 (27.5)	
No	771	328 (61.2)	443 (72.5)	
**Presentation and outcome**				
Hypoxic pneumonia (1⁰ outcome)				0.008
Yes	739	470 (34.1)	269 (40.1)	
No	1308	907 (65.9)	401 (59.8)	
PNG-defined severe pneumonia				<0.001
Yes	281	249 (18.1)	32 (4.8)	
No	1766	1128 (81.9)	638 (95.2)	
WHO-defined severe pneumonia^5^				<0.001
Yes	724	538 (39.1)	186 (27.8)	
No	1323	839 (60.9)	484 (72.2)	
Hospitalised				<0.001
Yes	623	544 (39.5)	79 (11.8)	
No	1424	833 (60.5)	591 (88.2)	
In-hospital death				0.37
Yes	12	12 (2.2)	-	
No	611	532 (97.8)	79 (100.0)	

IQR, interquartile range.

Note; denominators may vary due to missing data

1 Fully vaccinated: 0–11 months ≥2 doses of 13vPCV; ≥12 months ≥1 dose of 13vPCV. Under-vaccinated: 0–11 months ≤1 dose of 13vPCV; ≥12 months 0 doses of 13vPCV.

2 Prior to the national program, a small number of PNG children were enrolled in 10v/13vPCV trials.

3 Wet season refers to period from December to April inclusive, dry season refers to period from May to November inclusive.

4 Only available for the years 2016–2019.

5 Estimated based on respiratory rate and reported presence of WHO defined danger signs.

6 assessed by Chi-squared tests and Fisher's exact test (when *n*< 5).

Analysis of the raw data revealed that fully vaccinated children more frequently presented with hypoxic pneumonia (269/670; 40.1%) compared with under-vaccinated children (470/1377; 34.1%; OR: 1.29 [95% 10.7; 1.57]; Table 2). All other endpoints were less frequently observed in fully vaccinated children: PNG-defined severe pneumonia (32/670; 4.8% vs 249/1377; 18.1%), WHO-defined severe pneumonia (186/670; 27.8% vs 538/1377; 39.1%), hospitalisation (79/670; 11.8% vs 544/1377; 39.5%) and death (no in-hospital deaths occurred in vaccinated children compared with 12 episodes in unvaccinated children).

When adjusted for confounders (by propensity score; see supplementary Table 2), VE against hypoxic pneumonia was 28.7% (95%CI: 9.9, 43.6%). VE was also demonstrated against PNG-defined severe pneumonia (64.8%; 43.7, 77.9%) and hospitalised pneumonia (57.4%; 38.0, 70.7%) but no effectiveness against WHO-defined severe pneumonia and in-hospital mortality was demonstrated (Table 3). Similar VE estimates were obtained including all covariates in the model rather than adjusting by propensity scores (Table 4). To explore the impact of (i) potential misclassification of hypoxic pneumonia due to oxygen use and (ii) biased VE estimates due to inclusion of years of low vaccine uptake, analyses were repeated: (i) excluding those with oxygen saturations >90% whilst receiving oxygen and (ii) excluding those enrolled in 2013 and 2014. Using these approaches VE against hypoxic pneumonia ranged from 23.6% (4.0, 39.2) to 31.7% (13.5, 46.1); PNG-defined severe pneumonia ranged from 50.6% (21.3, 69.0) to 52.1% (24.1, 69.8) and hospitalised pneumonia ranged from 45.9% (26.3, 60.2) to 56.0% (34.7, 70.3).

**Table 3:** Unadjusted and adjusted 13vPCV vaccine effectiveness (estimated using inverse probability of treatment weighting).

Outcome	Unadjusted odds ratio (95% confidence interval)	Unadjusted vaccine effectiveness	Adjusted odds ratio*	Adjusted vaccine effectiveness*
Hypoxic pneumonia	1.29 (1.07; 1.57)	-29.5% (-56.6; -7.0)	0.71 (0.56; 0.90)	28.7% (9.9; 43.6)
PNG-defined severe pneumonia	0.23 (0.16; 0.33)	77.3% (66.8; 84.5)	0.35 (0.22; 0.56)	64.8% (43.7; 77.9)
WHO-defined severe pneumonia	0.60 (0.49; 0.73)	40.1% (26.7; 51.0)	0.78 (0.60; 1.02)	21.7% (-2.3; 40.1)
Hospitalised pneumonia	0.20 (0.16; 0.27)	79.5% (73.5; 84.2)	0.43 (0.29; 0.62)	57.4% (38.0; 70.7)

⁎ Adjusted by age, sex, year of enrolment, season (wet; dry), distance to hospital (<1 h; 1 h or more), absence or presence of comorbid conditions, malnutrition (weight-for-age z-score <2SD).

**Table 4:** Sensitivity analyses unadjusted and adjusted vaccine effectiveness.

Outcome	2013–2019: 13vPCV vaccine effectiveness using standard logistic regression		2013–2019: 13vPCV vaccine effectiveness estimated using inverse probability of treatment weighting excluding non-hypoxic pneumonia cases where O_2_ sats measured whilst on oxygen		2015–2019: 13vPCV vaccine effectiveness estimated using inverse probability of treatment weighting	
	Adjusted OR* (95%CI)	Adjusted VE* (95%CI)	Adjusted OR* (95%CI)	Adjusted VE* (95%CI)	Adjusted OR* (95%CI)	Adjusted VE* (95%CI)
Hypoxic pneumonia	0.76 (0.61; 0.96)	23.6% (4.0; 39.2)	0.68 (0.54; 0.86)	31.7% † (13.5; 46.1)	0.76 (0.61; 0.96)	23.7% (4.1; 39.3)
Severe PNG pneumonia	0.49 (0.32; 0.76)	51.2% (24.5; 68.4)	0.49 (0.31; 0.79)	50.6% (21.3; 69.0)	0.48 (0.30; 0.76)	52.1% (24.1; 69.8)
Severe WHO pneumonia	0.85 (0.65; 1.10)	15.5% (-10.2; 35.2)	0.82 (0.62; 1.07)	18.3% (-7.3; 37.7)	0.88 (0.68; 1.13)	12.1% (-12.9; 31.6)
Hospitalised pneumonia	0.54 (0.40; 0.74)	45.9% (26.3; 60.2)	0.44 (0.30; 0.65)	56.0% # (34.7; 70.3)	0.49 (0.36; 0.68)	50.9% (32.4; 64.4)

⁎ Adjusted by age, sex, year of enrolment, season (wet; dry), distance to hospital (<1 h; 1 h or more), absence or presence of comorbid conditions, malnutrition (weight-for-age z-score <2SD).

† Sensitivity analysis (excl all cases where O2 sats were measured whilst on O2): Vaccine effectiveness: 27.9% [8.8; 43.4]).

# Sensitivity analysis (excl all cases where O2 sats were measured whilst on O2: Vaccine effectiveness: 54.4% [30.7; 70.1]).

## Discussion

Our study demonstrates the effectiveness of 13vPCV against a variety of severe pneumonia outcomes including hypoxic pneumonia for children under 5 years of age in PNG. There remains a paucity of data supporting 13vPCV use in LMICs, particularly in Asia and the Pacific. This study highlights the tremendous benefits of PCVs in childhood immunisation programs. Given the ongoing high burden of pneumonia and challenges in seeking health care in PNG, routine use of 13vPCV is expected to substantially reduce the burden of pneumonia and childhood mortality. These data provide the evidence required to support policy makers’ decisions with regards to inclusion in the immunisation schedule, promotion and use of 13vPCV in PNG and other LMICs.

Compared with previously published studies, similar vaccine effectiveness estimates have been demonstrated in PNG. In randomised controlled trials (RCTs) conducted in Africa, the US, Philippines and Finland, the efficacy of PCV against radiologically-confirmed childhood pneumonia was estimated to be 27% (95% CI: 15, 36%; *p* < 0.0001).^5^ The overall impact on all-cause mortality of these studies was estimated to be 11% (95% CI: -1, 21%; *p* = 0.08). Although radiological pneumonia is the “gold standard” for epidemiological studies, it could not be used as an outcome measure in this setting as so few children received x-rays due to equipment unavailability. More recently, Mackenzie et al. demonstrated a 57% (95% CI: 42 to 67%) and 72% (58–82%) decline in hypoxic pneumonia in children 2–11 months and 12–23 months, respectively, following introduction of PCVs in The Gambia along with a 43% (-8% to 70%) reduction in radiologically-confirmed pneumonia.^7^ McCollum *et al* reported a 47% (5–70%) reduction in hypoxic pneumonia and a 65% (46–77%) reduction in clinical pneumonia with danger signs following introduction of 13vPCV in Malawi.^8^ Finally, using similar methodology to this study, a study in the Lao People's Democratic Republic provided the first estimates of 13vPCV effectiveness against hypoxic pneumonia in Asia (Laos: 2014–2018; adjusted VE against hypoxic pneumonia: 35 [7–55%] to 37% [6–57%]).^9^

Despite this increasingly strong evidence and inclusion of PCVs in national immunisation programs in nearly 150 countries, PCVs have been included in the routine infant immunisation schedule in a limited number of countries in the WHO South-East Asia and Western Pacific Regions.^28^ Absence of programs in these populous countries including China, Thailand and Vietnam mean the majority of children in Asia do not receive the health and mortality benefits expected through routine PCV use. Strategies to overcome the financial and economic barriers, logistical obstacles and cultural challenges including vaccine hesitancy must be prioritised so that the protection offered by conjugate pneumococcal vaccination is available to all children and societies.^29^

Although PCV uptake appears to be improving in this cohort (up to 69% in children enrolled in this single centre cohort study), national estimates suggest that under-vaccination is common for PNG children. The proportion of districts achieving three dose diphtheria-tetanus-pertussis coverage greater than the WHO-recommended benchmark of 80% is only 8% in 2019, falling from 28% in 2014, although the accuracy of these estimates remains uncertain.^30^ Ongoing investment in the national immunisation program and strengthening of health systems more broadly must be prioritised, particularly during the current SARS-CoV-2 pandemic, if the expected benefits of 13vPCV are to be realised.

In addition to demonstrating the effectiveness of 13vPCV in PNG, this study further validates the method described by Weaver et al.^9^ to estimate vaccine effectiveness against hypoxic pneumonia. This method overcomes many of the limitations of traditional case-control studies which remain challenging to implement in real-world settings, particularly in LMICs. Drawing cases and controls from patients hospitalised for the same condition, enabled us to more reliably estimate vaccine effectiveness. Our results highlight the critical importance of controlling for confounders: the primary outcome was observed more frequently in vaccinated children yet the direction of the effect reversed when adjusted for covariates and year of enrolment (which was particularly influential in the model). These estimates appeared to remain robust using different analytical techniques and through sensitivity analyses. As demonstrated previously, the proportion of children with a defined clinical endpoints (e.g. severe pneumonia) is highly susceptible to subtle changes in case definition.^31^ Our results demonstrate that vaccine effectiveness point estimate may vary by the chosen clinical endpoint, highlighting the benefit of assessing multiple outcomes and inclusion of outcomes, such as hypoxic pneumonia, which can be more consistently applied across populations.

This is the first estimate of PCV effectiveness in PNG. The strengths of this study include the prospective recruitment of more than 2000 pneumonia cases using a standard approach over a 6 year period and low-to-moderate vaccination coverage which limits the impact of any indirect effects on our findings. High rates of data capture including both oxygen saturations and immunisation status were achieved. There are a number of limitations to the study. We were reliant on hand-held records to assess vaccine status and so underestimation of PCV vaccine coverage may have occurred.^19^ Recruitment was undertaken from sites within Goroka, potentially limiting the generalisability of the findings. Enrolled cases were a subset of the 6316 pneumonia admissions to EHPH and many more outpatient presentations during the study period.^12^ Inclusion criteria were aligned with the PNG clinical manual meaning that secondary endpoints including WHO-defined severe pneumonia needed to be estimated in a post-hoc fashion, potentially decreasing the accuracy of VE estimates against this clinical endpoint. The primary reason for hospital admission was not recorded with those with pneumonia admitted for other reasons indistinguishable from those with pneumonia-related admissions. Additional covariates including breastfeeding, dwelling type, number of household residents and household smoke exposure were not collected in those enrolled before 2016, limiting our ability to control for these covariates for all children in this study.

In conclusion, we demonstrate that two doses of 13vPCV in PNG children aged 0-11 months and/or one dose in those older than 12 months was associated with a 29% reduction in hypoxic pneumonia, a 65% reduction in severe pneumonia (as per PNG definitions) and a 57% reduction in pneumonia hospitalisation. We were unable to determine an impact against in-hospital mortality. Strategies to improve vaccine coverage and increase access to 13vPCV in PNG and similar LMICs are urgently required. Ongoing surveillance and regular estimates of vaccine effectiveness against clinical endpoints are required given the selective pressure expected to be observed for serotypes not included in the vaccine. This will be essential to inform potential use of extended valency PCVs expected to be available in the future.

### Contributors

CCB was the PNG lead investigator for both studies and led the write up of this manuscript. KJB led statistical analysis with the assistance of CCB and CDN. JS, JK, BN and WK led recruitment and data management. RF, DL and WP led the design and on-site supervision of the first study. FMR was the lead researcher for the three country PneuCAPTIVE study. JC, CS, RF and WP assisted with the design and implementation of the PneuCAPTIVE study. All authors reviewed the manuscript prior to publication.

### Data sharing statement

The study protocol and informed consent form are available upon request. Individual data from this study has not been made publicly available, as data are only approved for use for the purposes outlined in the study protocol. Requests for de-identified data are subject to approval by the Papua New Guinea Institute of Medical Research Institutional Review Board and PNG Medical Research Advisory Committee. We recommend that requests for data also be sent to Professor Chris Blyth.

## Declaration of interests

Investigators are supported by National Health and Medical Research Council Investigator grants and fellowships (CCB; CS; FMR). The studies from which data were derived were funded by Pfizer Global and the Bill & Melinda Gates Foundation. CDN and CS receive grants from Pfizer, outside the submitted work.

## Appendix Supplementary materials

Image, application 1
