# Supplementary material for: Effectiveness of 13-valent pneumococcal conjugate vaccine against hypoxic pneumonia and hospitalisation in Eastern Highlands Province, Papua New Guinea: An observational cohort study
Source: Lancet Reg Health West Pac. 2022 Mar 15;22:100432. doi: 10.1016/j.lanwpc.2022.100432 (PMC8927990; doi:10.1016/j.lanwpc.2022.100432)
Supplement: Supplementary file 1 [file mmc1.docx]

**Supplement table 1: STROBE checklist**

STROBE Statement—Checklist of items that should be included in reports of ***cohort studies***

|  | Item No | Recommendation | Response |  |
| --- | --- | --- | --- | --- |
| **Title and abstract** | 1 | (*a*) Indicate the study’s design with a commonly used term in the title or the abstract: | Included |  |
|  |  | (*b*) Provide in the abstract an informative and balanced summary of what was done and what was found | Included |  |
| Introduction | | |  |  |
| Background/rationale | 2 | Explain the scientific background and rationale for the investigation being reported | Included |  |
| Objectives | 3 | State specific objectives, including any prespecified hypotheses | Included |  |
| Methods | | |  |  |
| Study design | 4 | Present key elements of study design early in the paper | Included |  |
| Setting | 5 | Describe the setting, locations, and relevant dates, including periods of recruitment, exposure, follow-up, and data collection | Included |  |
| Participants | 6 | (*a*) Give the eligibility criteria, and the sources and methods of selection of participants. Describe methods of follow-up | Included |  |
|  |  | (*b*) For matched studies, give matching criteria and number of exposed and unexposed | Included |  |
| Variables | 7 | Clearly define all outcomes, exposures, predictors, potential confounders, and effect modifiers. Give diagnostic criteria, if applicable | Included |  |
| Data sources/ measurement | 8* | For each variable of interest, give sources of data and details of methods of assessment (measurement). Describe comparability of assessment methods if there is more than one group | Included |  |
| Bias | 9 | Describe any efforts to address potential sources of bias | Included |  |
| Study size | 10 | Explain how the study size was arrived at | Included |  |
| Quantitative variables | 11 | Explain how quantitative variables were handled in the analyses. If applicable, describe which groupings were chosen and why | Included |  |
| Statistical methods | 12 | (*a*) Describe all statistical methods, including those used to control for confounding | Included |  |
|  |  | (*b*) Describe any methods used to examine subgroups and interactions | Included |  |
|  |  | (*c*) Explain how missing data were addressed | N/A |  |
|  |  | (*d*) If applicable, explain how loss to follow-up was addressed | Included |  |
|  |  | (*e*) Describe any sensitivity analyses | Included |  |
| Results | | |  |  |
| Participants | 13* | (a) Report numbers of individuals at each stage of study—eg numbers potentially eligible, examined for eligibility, confirmed eligible, included in the study, completing follow-up, and analysed | Included |  |
|  |  | (b) Give reasons for non-participation at each stage | Included |  |
|  |  | (c) Consider use of a flow diagram | Included |  |
| Descriptive data | 14* | (a) Give characteristics of study participants (eg demographic, clinical, social) and information on exposures and potential confounders | Included |  |
|  |  | (b) Indicate number of participants with missing data for each variable of interest | N/A |  |
|  |  | (c) Summarise follow-up time (eg, average and total amount) | N/A |  |
| Outcome data | 15* | Report numbers of outcome events or summary measures over time | Included |  |
| Main results | 16 | (*a*) Give unadjusted estimates and, if applicable, confounder-adjusted estimates and their precision (eg, 95% confidence interval). Make clear which confounders were adjusted for and why they were included | Included |  |
|  |  | (*b*) Report category boundaries when continuous variables were categorized | Included |  |
|  |  | (*c*) If relevant, consider translating estimates of relative risk into absolute risk for a meaningful time period | N/A |  |
| Other analyses | 17 | Report other analyses done—eg analyses of subgroups and interactions, and sensitivity analyses | Included |  |
| Discussion | | |  |  |
| Key results | 18 | Summarise key results with reference to study objectives | Included |  |
| Limitations | 19 | Discuss limitations of the study, taking into account sources of potential bias or imprecision. Discuss both direction and magnitude of any potential bias | Included |  |
| Interpretation | 20 | Give a cautious overall interpretation of results considering objectives, limitations, multiplicity of analyses, results from similar studies, and other relevant evidence | Included |  |
| Generalisability | 21 | Discuss the generalisability (external validity) of the study results | Included |  |
| Other information | | |  |  |
| Funding | 22 | Give the source of funding and the role of the funders for the present study and, if applicable, for the original study on which the present article is based | Included |  |

**Supplemental table 2: percentage standardised differences in covariates before and after inverse probability of treatment weighting**

| **Characteristics** | **Standardised difference**  **Pre-IPTW** | **Standardised difference**  **Post-IPTW** |
| --- | --- | --- |
| Age (months) | -6.70 | 4.86 |
| Sex | 1.49 | -3.24 |
| Season | -0.70 | 3.13 |
| Distance to hospital | -32.11 | -5.69 |
| Comorbidities | 9.54 | 5.33 |
| Malnutrition | 16.83 | -1.66 |
